# Quality of life, depression and anxiety among pregnant women with previous adverse pregnancy outcomes

**DOI:** 10.1590/S1516-31802009000400002

**Published:** 2009-12-07

**Authors:** Evelyn Regina Couto, Egle Couto, Bruna Vian, Zoraide Gregório, Marcelo Luis Nomura, Renata Zaccaria, Renato Passini

**Affiliations:** 1 MSc. Physiotherapist, Department of Obstetrics and Gynecology, Faculdade de Medicina da Universidade Estadual de Campinas (FCM/Unicamp), Campinas, São Paulo, Brazil.; 2 MD, PhD. Obstetrician and Gynecologist, Department of Obstetrics and Gynecology, Faculdade de Medicina da Universidade Estadual de Campinas (FCM/Unicamp), Campinas, São Paulo, Brazil.; 3 Obstetric nurse, Department of Obstetrics and Gynecology, Faculdade de Medicina da Universidade Estadual de Campinas (FCM/Unicamp), Campinas, São Paulo, Brazil.; 4 MD. Obstetrician and Gynecologist, Department of Obstetrics and Gynecology, Faculdade de Medicina da Universidade Estadual de Campinas (FCM/Unicamp), Campinas, São Paulo, Brazil.

**Keywords:** Quality of life, Depression, Anxiety, Pregnancy, Reproductive history, Qualidade de vida, Depressão, Ansiedade, Gravidez, História reprodutiva

## Abstract

**CONTEXT AND OBJECTIVE:**

Previous adverse pregnancy outcomes (recurrent spontaneous abortion, fetal death, preterm birth or early neonatal death) can affect the quality of life of pregnant women. The objective of this study was to compare the quality of life and the prevalence of symptoms of anxiety and depression among pregnant women with and without these antecedents.

**DESIGN AND SETTING:**

An analytical cross-sectional study was performed in four settings (two high-risk and two low-risk prenatal clinics) in the city of Campinas, São Paulo, Brazil.

**METHODS:**

A total of 240 women were interviewed by a single investigator between the 18^th^ and 24^th^ weeks of gestation: 120 women with prior adverse pregnancy outcomes (group 1) and 120 women with no such history (group 2), matched according to their numbers of living children. Sociodemographic variables were collected and two questionnaires were used: the Short Form-36 quality-of-life questionnaire and the Depression and Anxiety Scale.

**RESULTS:**

The women in group 1 had lower scores in all the items on the quality-of-life questionnaire. Depression and anxiety were more frequent in group 1 (P < 0.0001). An inverse correlation was found between the Short Form-36 domains and anxiety and depression.

**CONCLUSIONS:**

Women with histories of recurrent spontaneous abortion, fetal death, preterm birth or early neonatal death seem to have poorer quality of life and more symptoms of anxiety and depression during their subsequent pregnancy, compared with those without such antecedents.

## RESUMO

**CONTEXTO E OBJETIVO:**

O antecedente de resultados gestacionais adversos (aborto espontâneo recorrente, óbito fetal, prematuridade ou óbito neonatal precoce) pode afetar a qualidade de vida das gestantes. O objetivo deste estudo foi comparar a qualidade de vida e a prevalência de sintomas de ansiedade e depressão em gestantes com e sem estes antecedentes.

**TIPO DE ESTUDO E LOCAL:**

Um estudo transversal analítico foi realizado em quatro locais (duas clínicas de pré-natal de alto risco e duas de pré-natal de baixo risco), na cidade de Campinas, São Paulo, Brasil.

**MÉTODOS:**

Duzentas e quarenta mulheres foram entrevistadas por um único investigador entre 18 e 24 semanas de gestação: 120 tinham antecedentes gestacionais adversos (grupo 1) e 120 não tinham tal história (grupo 2), pareadas por número de filhos vivos. Variáveis sócio-demográficas foram coletadas e dois questionários foram usados: o de qualidade de vida (Questionário Short Form-36) e a Escala de Depressão e Ansiedade.

**RESULTADOS:**

As mulheres do grupo 1 obtiveram escores mais baixos em todos os itens do questionário de qualidade de vida. Depressão e ansiedade foram mais frequentes no grupo 1 (P < 0,0001). Foi encontrada uma correlação inversa entre os domínios do Short Form-36 e ansiedade e depressão.

**CONCLUSÕES:**

Mulheres com antecedente de aborto espontâneo recorrente, óbito fetal, prematuridade ou óbito neonatal precoce parecem ter pior qualidade de vida e mais sintomas de ansiedade e depressão durante a gestação subsequente quando comparadas com mulheres sem esses antecedentes.

## INTRODUCTION

Several emotional and physical changes occur during pregnancy. One study on the impact of prior pregnancy loss on subsequent pregnancy revealed high levels of anguish: the principal symptom reported was a mixture of hope and fear.^1^ Fetal and neonatal losses are stressful events that may lead to serious long-term effects.^2^ Traumatic experiences involve a pattern of psychological and physiological reactions such as anxiety, depression, irritability, excess fatigue, sleep disorders and concentration difficulties.^3^ Denial and repression of feelings may lead to a greater likelihood of adverse health effects and costly disorders such as posttraumatic stress.^4^ Various studies have described high rates of symptoms of anxiety and depression following perinatal loss.^1^^,^^5^^,^^6^ Nevertheless, little is known of the consequences of continuous grief on future pregnancies.^1^ Few studies have evaluated quality of life during pregnancy.

In order to develop interventions to help reduce psychological stress, it is important to understand the effect of this type of loss on the parents. Evaluating the extent to which the trauma of adverse pregnancy outcomes is an event that may trigger psychological disorders in women during subsequent pregnancy is the first step.^1^

## OBJECTIVE

The objective of this study was to evaluate the quality of life and frequency of symptoms of anxiety and depression among women with histories of adverse pregnancy outcomes (recurrent spontaneous abortion, fetal death, preterm birth or neonatal death). The objective outlined here was based on the following question: Do women with histories of adverse pregnancy outcomes have poorer quality of life and more symptoms of anxiety and depression in subsequent pregnancy than women with no such history?

## METHODS

Between 2005 and 2006, an analytical cross-sectional study was performed in four settings in the city of Campinas, Brazil: a university clinic for high-risk pregnancies (Centro de Atenção Integral à Saúde da Mulher, Universidade Estadual de Campinas; CAISM/Unicamp), a tertiary hospital (Campinas Maternity Hospital) and two municipal healthcare clinics offering prenatal care to low-risk women (Centro de Saúde São José and Centro de Saúde Taquaral). This study was approved by the Internal Review Board of Unicamp.

The inclusion criteria were that the women should be 15-40 years of age and present a gestational age of 18-24 weeks. This gestational age was chosen so that the discomforts of the first trimester had disappeared (nausea or vomiting) and those of late pregnancy had not yet appeared (backache, lower limb edema or breathing difficulties). The exclusion criteria were that the women should not present multiple pregnancy, polyhydramnios, morbid obesity or histories of mental disease. Women with the first three of these exclusion conditions could be at high risk of preterm birth or have stronger low back pain,^7^^,^^8^^,^^9^ thereby modifying the quality-of-life evaluation.

Group 1 consisted of 120 pregnant women with a history of one or more of the following adverse pregnancy outcomes: recurrent abortion (≥ 3 spontaneous successive fetal losses before 20 weeks,^10^ fetal death (death of a product of conception prior to complete expulsion or extraction from its mother, irrespective of the duration of pregnancy^11^), preterm birth (delivery before 37 weeks of gestation^11^) or early neonatal death (death of a live-born baby within the first seven days of life^11^). Group 2 consisted of 120 pregnant women without any of the aforementioned adverse outcomes, matched for their numbers of living children. 

The women in group 1 were receiving special antenatal care at the high-risk clinic or at the clinic of the tertiary hospital, while the women in group 2 were receiving routine prenatal care at the two primary care units.

On the same day as the women’s routine prenatal consultations, all their medical and nursing records were evaluated by the first author to check whether they had any histories of the predefined adverse pregnancy outcomes and whether they met the inclusion criteria. The women who fulfilled the criteria were invited by the investigator to participate voluntarily in the study. All those who agreed signed an informed consent form and were interviewed orally using a structured questionnaire.

Each interview took about 20 minutes and was carried out in a private office. Initially, the investigator collected sociodemographic variables: maternal age, race (self-reported), marital status, number of previous pregnancies, type of delivery, abortions, education, family income, alcoholism (disease and addiction that resulted in persistent use of alcohol despite the negative consequences) and drug addiction (progression from acute drug use to the development of drug-seeking behavior, vulnerability to relapse and decreased, slowed ability to respond to naturally rewarding stimuli). After this, the Short Form-36 (SF-36) and Hospital Anxiety and Depression Scale (HADS) questionnaires were applied.

The Health Insurance Experience (HIE) and Medical Outcomes Study (MOS) instruments were historical milestones in the evaluation of quality of life.^12^ The MOS gave rise to the SF-36 questionnaire. The SF-36 is a generic instrument consisting of eight areas divided into two components, i.e. physical and mental health summary scales.^13^ In Brazil, the SF-36 was translated and validated in 1999, and it has been used in various studies that evaluated patients with rheumatoid arthritis,^13^ coronary disease,^14^ chronic renal failure^15^ and endometriosis.^16^ The HADS was originally developed to evaluate the psychological stress of parents in medical and surgical environments.^17^^,^^18^^,^^19^ In Brazil, the HADS was validated in 1995.^20^

The SF-36 has been used to evaluate perceived quality of life in the areas of physical and mental health.^21^ It includes evaluations of eight health domains: physical functioning, physical role limitations, bodily pain, general health, vitality, social functioning, emotional role limitations and mental health. The measurements are generic and serve to compare patients presenting chronic health problems with individuals from the general population. Previous studies have confirmed the reliability and validity of the SF-36 for use in sampled patient populations or in the general population. In the present study, the SF-36 scores were obtained in accordance with the previously described procedures.^21^ In final evaluations, a number from 0 to 100 is attributed to each question, 0 representing the worst state of health and 100 the best. In order to avoid false interpretations, there is no value that can summarize the total evaluation, since each score is an isolated calculation.^22^

HADS is a tool widely used to detect clinically significant cases of anxiety or depression. It contains 14 items in two subscales: seven items referring to anxiety and seven to depression. The scores are marked on a four-point scale that ranges from 0 (not at all or only occasionally) to 3 (most of the time or a great deal of the time). The scores of the subscales are added together, resulting in overall scores of 0-21 for the HADS-A and HADS-D subscales. Scores of 11 or more in each subscale are considered indicative of psychological morbidity (clinical cases), whereas scores between 8 and 10 are indicative of mood disorders.

There were no data available in the literature concerning adverse pregnancy outcomes and SF-36. For this reason, the difference in mean SF-36 scores between depressed and non-depressed pregnant women^23^ was used to calculate the sample size. The calculation was based on Student’s t test, with a 5% significance level and an 80% test power (a = 0.05 and b = 0.20). The minimum sample size obtained was 158 women (total) which was sufficient to find significant differences for all the items of the SF-36.

The average number of pregnant women receiving prenatal care during the study period, at each site from which cases and controls were obtained, was around 300.

### Data analysis

The results from the SF-36 were evaluated and expressed as means, standard deviations, medians and ranges for each group, and the two groups were compared using the Wilcoxon signed-rank test. The results from the HADS questionnaire were evaluated in each group using the chi-square test. The significance level was defined as 5% throughout the entire statistical analysis, which was carried out using the Statistical Analysis System (SAS) software package, version 8.2.

## RESULTS

A total of 258 women were invited to participate in the study and 18 of them refused for various reasons: 15 said that they did not have time to take part (ten in group 2 and five in group 1) and three (all in group 1) refused without specifying a motive.

As presented in Table 1, there were significant differences between the two groups regarding age (30.3 versus 27.6 years), smoking (4% versus 25%), hypothyroidism (22% versus 3%), mean number of previous pregnancies (3.7 versus 1.4) and mean number of previous deliveries (1.2 versus 0.4) (P < 0.0001 for all the variables).

The period between last delivery and the index pregnancy ranged from 12 to 36 months for both groups, with means of 23.6 and 22.8 months for groups 1 and 2, respectively. In group 1, there were 14 women with a history of recurrent abortion, 49 with preterm deliveries and five with early neonatal deaths. Fifty-two women had histories of more than one adverse event: 36 had had three or more miscarriages, 14 had had more than one premature delivery and two had had more than one previous fetal death.

The SF-36 scores for each variable are shown in Table 2. Statistically significant differences were found between the two groups for each of the variables evaluated. There were significantly more women with symptoms of anxiety and depression in group 1 than in group 2 (Table 3).

**Table 1.** Main characteristics of participants

	Group 1 Previous adverse pregnancy outcomes	Group 2 Controls	P
Age in years: mean (SD)	30.3 (5.9)	27.6 (5.9)	< 0.0001
Number of previous pregnancies: mean (SD)	3.7 (1.6)	1.4 (0.6)	−
Number of previous deliveries: mean (SD)	1.2 (1.0)	0.4 (0.7)	−
Married*	88	75	NS
White race	73	70	NS
Education > 10 years	32	46	NS
Monthly income up to 292 United States dollars	72	70	NS
Smoking	4	25	< 0.0001
Alcoholism	4	5	NS
Drug addiction	1	3	NS
Diabetes mellitus	8	5	NS
Hypertension	12	15	NS
Hypothyroidism	22	3	< 0.0001

All values expressed as percentages of 120 patients unless otherwise stated. *Legal or common law marriage. SD = standard deviation; NS = not significant.

**Table 2.** Quality of life (Short Form-36) among women with previous adverse pregnancy outcomes (group 1) and controls (group 2)

	Group 1 (adverse outcomes)		Group 2 (controls)		P
	Mean (SD)	Median	Mean (SD)	Median	
Physical functioning	52.7 (29)	55	74.3 (18.8)	75	< 0.0001
Physical role limitations	31.5 (35.3)	25	48.3 (39.9)	50	< 0.0001
Body pain	50.7 (22.8)	41.5	67.1 (23)	62	< 0.0001
General health	63.4 (24.7)	67	87.8 (15.3)	92	< 0.0001
Vitality	48 (21)	45	59.2 (22.6)	65	< 0.0001
Social functioning	56.4 (27.5)	50	67.1 (24.4)	62.5	0.0013
Emotional role limitations	36.9 (38.6)	33.3	63.3 (39.7)	66.7	0.0233
Mental health	55 (21.5)	56	71.2 (20.9)	76	< 0.0001
Anxiety	10.1 (3.9)	11	6.2 (3.7)	5.5	< 0.0001
Depression	6.3 (3.1)	6	3.9 (2.8)	3	< 0.0001
Number of cases	120		120		

Wilcoxon test, matched according to number of live children. SD = standard deviation.

**Table 3.** Anxiety and depression (Hospital Anxiety and Depression Scale) among women with previous adverse pregnancy outcomes (group 1) and controls (group 2)

Scores ≥ 8	Group 1 (adverse outcomes)		Group 2 (controls)		P^*^
	n	%	n	%	
Anxiety	92	76.7	40	33.3	< 0.0001
Depression	39	32.5	12	10.0	< 0.0001

*Chi-squared test.

## DISCUSSION

It is impossible to fully comprehend the impact of adverse pregnancy outcomes on the subsequent pregnancy. However, some measurements may be able to show the effect of these outcomes on quality of life. Some of the consequences of previous pregnancy loss on the subsequent pregnancy are reflected in the results from the SF-36 domains. A study that investigated the psychometric properties of the SF-36 questionnaire in relation to the index pregnancy confirmed that this tool could be used in clinical practice to measure quality of life on eight subscales during early pregnancy.^24^

In our study, women with adverse pregnancy histories had poorer results in all the items evaluated (quality of life, anxiety and depression). Fetal death, repeated spontaneous abortion, preterm deliveries and early neonatal deaths represent abrupt interruptions of personal and family adaptations to pregnancy and demand new adaptations to an unexpected situation. These events can generate anxiety during future pregnancies and affect the parents’ quality of life. Psychological and emotional stress and fatigue were identified as factors that compromised the quality-of-life domains.^25^

Perinatal loss has long-term effects.^1^ Pregnancy loss occurs at a time at which a new life is expected, and there may be no visible child, memories or shared experiences. Moreover, society may not recognize the significance of this type of loss for the parents.^26^ Several investigators have described symptoms consistent with psychological disorders among parents with histories of previous perinatal loss.^1^^,^^27^ Approximately half of mothers report high levels of symptoms of depression, thus placing them at high risk of depression.^28^ Reports in the literature show that the prevalence of stress and symptoms of depression and anxiety are higher during pregnancy than during other periods of life, particularly among vulnerable populations.^29^ An association between symptoms of anxiety and depression during pregnancy and adverse pregnancy outcomes such as preterm birth and low birth weight has been described in previous publications.^30^^,^^31^ In the present study, women with histories of recurrent abortion, fetal death and early neonatal death had lower scores for physical functioning and greater body pain than women without this type of history. They also had high rates of symptoms of depression. Physical symptoms were found to be associated with occurrences of depression and anxiety during pregnancy, thus leading to the suggestion that affective disorders may increase somatic symptoms.^25^ Few studies to this date have evaluated the relationships between anxiety, depression and quality of life among pregnant women. Armstrong and Hutti found greater rates of anxiety during pregnancy among women who had had a previous perinatal loss.^28^

Appraising the parents’ emotions during a pregnancy subsequent to a perinatal loss may lead to a better understanding of the needs of these families at critical moments.^28^ In a study on parents following a perinatal loss or sudden infant death, Vance et al. reported that a gradual reduction in the symptoms of depression and anxiety occurred over time. However, even 30 months after the loss, the parents continued to have almost twice as much psychological stress as shown by the parents in the control group.^27^ In addition, the current findings show that there is no relationship between parents’ psychological stress during a pregnancy subsequent to a perinatal loss and the development of their relationship with the fetus of the index pregnancy.^1^ Nevertheless, qualitative comments in another study suggest that some parents attempt to delay the onset of their relationship with the baby.^1^^,^^28^ It is important to evaluate the early development of relationships between parents and the fetus/newborn infant, in order to identify the effect of prior pregnancy loss on this relationship.^1^

The recognition that anxiety and depression during pregnancy may be associated with adverse outcomes has led to a search for stress-reducing interventions, with promising results.^32^^,^^33^ Managing psychological stress may affect the course of the current pregnancy, as well as the future relationship between the parents and the child.^1^

## CONCLUSIONS

Women with histories of recurrent spontaneous abortion, fetal death, preterm birth or early neonatal death seem to have poorer quality of life and greater symptoms of anxiety and depression during their subsequent pregnancy than do those without these adverse pregnancy outcomes.
